# Kinetic modeling of ^18^F-PI-2620 binding in the brain using an image-derived input function with total-body PET

**DOI:** 10.1186/s13550-025-01260-4

**Published:** 2025-05-30

**Authors:** Anjan Bhattarai, Emily Nicole Holy, Yiran Wang, Benjamin A. Spencer, Guobao Wang, Charles DeCarli, Audrey P. Fan

**Affiliations:** 1 Department of Neurology, University of California, Davis, 1590 Drew Avenue, Unit #100, CA 95618 Davis, USA; 2 Department of Biomedical Engineering, University of California, Davis, Davis, CA USA; 3 Department of Radiology, University of California, Davis, Davis, CA USA

**Keywords:** Tau, ^18^F-PI-2620, Kinetic modeling, Grey matter, Image-derived input function, Total-body PET

## Abstract

**Background:**

Accurate quantification of tau binding from ^18^F-PI-2620 PET requires kinetic modeling and an input function. We aimed to implement a non-invasive Image-derived input function (IDIF) using the state-of-the-art total-body uEXPLORER PET/CT scanner to quantify tau binding and tracer delivery rate from ^18^F-PI-2620 in the brain. Additionally, we investigated the impact of scan duration on the quantification of kinetic parameters.

**Results:**

^18^F-PI-2620 total-body PET dynamic (90 min) data from 15 elderly (66–92 years) participants were acquired. Time-activity curves were obtained from grey matter regions of interest (ROIs) known to be affected in Alzheimer’s disease, including the medial temporal lobe, posterior cingulate, and lateral parietal cortex. These curves were fitted to the two-tissue compartmental model (2TCM) using a subject-specific IDIF (plasma and metabolite corrected) derived from the descending aorta. ROI-specific kinetic parameters were estimated for different scan durations ranging from 10 to 90 min. The parameters included blood fraction volume (v_b_), rate constants (K_1_, k_2_, k_3_, k_4_), total distribution volume (V_T_), distribution volume ratio (DVR), and tracer arrival delay. Logan graphical analysis was also used to estimate V_T_ and compared with 2TCM. Differences in kinetic parameters were observed between ROIs, including significant reduction in tracer delivery rate (K_1_) in the medial temporal lobe (q < 0.001). All kinetic parameters remained relatively stable (compared to parameters quantified with full 90-minute data) after the 60-minute scan window across all ROIs (*r* ≥ 0.89; *p* < 0.001), with K_1_ showing high stability after 30 min of scan duration (*r* ≥ 0.92; *p* < 0.001). Excellent correlation was observed between V_T_ estimated using 2TCM and Logan plot analysis (*r* ≥ 0.96; *p* < 0.001).

**Conclusions:**

This study demonstrated the utility of IDIF from a lager blood pool, derived using the total-body PET in quantifying ^18^F-PI-2620 kinetics in the brain. Our findings suggest that a 60-minute scan window may be required for the reliable quantification of kinetic parameters using IDIF, whereas a 30-minute scan time may be sufficient for the quantification of K_1_.

**Supplementary Information:**

The online version contains supplementary material available at 10.1186/s13550-025-01260-4.

## Introduction

^18^F-PI-2620 is a second generation PET radiotracer that has high binding affinity for tau protein aggregates in the brain, a key pathological feature in Alzheimer's disease (AD), progressive supranuclear palsy, and other taopathies [1–4]. Tau overload in the brain has been suggested to play a role in synaptic degeneration and neuronal loss [5]. Initial clinical investigations and visual assessments have shown increased ^18^F-PI-2620 binding in the brain in individuals with AD [4, 6]. However, there is a need for studies investigating subtle change in tau binding in early-stage AD and elderly individuals at risk of AD. Accurate, non-invasive quantification of ^18^F-PI2620 tau load in the brain is critical in research as well as clinical trials with therapeutic interventions.

Conventionally, standard uptake value ratio (SUVR) has been used to evaluate tracer distribution in the brain [6–8]. SUVR is a semiquantitative measure and is normalized to tracer uptake in a reference region, e.g. the inferior cerebellum for tau PET. SUVR comparisons across disease groups assume that tracer uptake in a reference region remains unchanged despite the presence of pathologies. SUVR does not take into account other pharmacokinetic components, such as blood volume, tracer metabolism, and blood flow, which may influence the overall uptake in the targeted region. Furthermore, SUVR does not characterize the transport information of the tracer.

Accurate quantification of tau binding from ^18^F-PI-2620 PET requires kinetic modeling and an input function. Conventionally, the arterial input function (AIF), which requires arterial blood sampling, has been used for kinetic modeling [9]. Although AIF is considered the gold-standard approach for kinetic parameter estimation, it is an invasive approach and may cause discomfort and discourage participation in studies. AIF is also susceptible to sampling errors, delay, and dispersion, which may often lead to inaccurate estimation of kinetic parameters. Non-invasive image-derived input function (IDIF), derived using dynamic PET imaging, has emerged as an alternative to AIF [9–11]. IDIF eliminates the need for arterial cannulation, manual blood handling, and analyses, thereby minimizing patient discomfort and reducing the exposure of additional radiation to the research personnel involved in data acquisition [11]. It is generally challenging to obtain a reliable IDIF for dynamic brain PET imaging with a conventional short scanner due the limited axial field-of-view that does not cover a major blood pool [12].

A recent development in PET imaging, the total-body uEXPLORER PET/CT scanner, offers an opportunity to generate total-body dynamic images with its long axial field-of-view, high photon collection efficiency, and high spatial resolution [13, 14]. More importantly, it enables us to extract IDIF from large blood pools such as the aorta and ventricles, which are less susceptible to partial volume effects compared to the conventionally suggested carotid artery. Our previous amyloid study with total-body PET demonstrated the utility of descending aorta IDIF for quantifying grey matter kinetics in the brain [15].

Kinetic modeling with ^18^F-PI-2620 requires dynamic PET data with relatively longer scan time (generally over one hour). However, such long scanning durations are cost-intensive and can be challenging for elderly participants. A previous study suggested that a shorter scan time (i.e., 40 min) can be sufficient for the measurements of semi quantitative SUVR in the brain in individuals with progressive supranuclear palsy (PSP) [16]. There is a need for studies investigating the sensitivity and efficacy of shorter scan durations for estimating ^18^F-PI-2620 grey matter kinetics, quantified through a compartmental modeling approach, particularly in individuals with AD or those at risk of developing it.

Earlier findings have identified regional differences in ^18^F-PI-2620 uptake in AD cohort [4, 6]. The medial temporal lobe, including the hippocampus, entorhinal cortex, and amygdala, has been reported to have higher tau accumulation in AD [6]. Tau kinetics in the grey matter regions known to be impacted based on Braak staging [17], in elderly cohorts at risk of AD, warrant further investigation. Investigating early disease stage tau kinetics with specific pharmacokinetic components is critical in research, as well as for clinical trials aimed at therapeutic intervention to delay disease progression.

This study aimed to implement the IDIF derived from a major blood pool using the total-body uEXPLORER PET/CT scanner to quantify ^18^F-PI-2620 kinetics with kinetic modeling of key brain grey matter regions in elderly individuals. Additionally, we investigated how scan duration influences the estimation of micro and macro kinetic parameters across grey matter region of interest (ROIs), quantified through two-tissue compartment modeling (2TCM). Furthermore, total distribution volume (V_T_), estimated using 2TCM was compared to the simpler graphical Logan plot analysis, both using IDIF. Finally, the study aimed to explore differences in the kinetic parameters across ROIs.

## Materials and methods

### Participants

Ethics approval for this study was obtained from the UC Davis Institutional Review Board. All the participants recruited in the study were part of the UC Davis Alzheimer’s Disease Research Center, over 65 years of age and gave written informed consent. Eligibility criteria included the ability to undergo an MRI and a known cognitive status based on clinical assessment and neuropsychological testing. Individuals with pacemakers, brain tumors, alcoholism, and/or those who were not able to lie still for 90 min were not included in this study. The study cohort included 15 individuals (age = 66–92 years; male = 9; female = 6), including 11 cognitively unimpaired individuals, 2 individuals with mild cognitive impairment, and 2 with Alzheimer’s disease. Our cohort included 2 amyloid positive participants but did not include tau positive individuals. Tau and amyloid status were determined by a clinical read on static amyloid and tau PET scans by a trained neurologist (C.D.) with over 30 years of experience.

### Data acquisition

#### PET

Dynamic total-body ^18^F-PI-2620 PET images were acquired for 90 min using the uEXPLORER and reconstructed with ordered-subset expectation-maximization (OSEM), (resolution = 2.344 mm isotropic voxels, four iterations, 20 subsets, with all standard data corrections except resolution modelling, and no post-reconstruction smoothing, following the UC Davis clinical protocol [18]. A high temporal resolution dynamic framing protocol was used: 30 × 2s, 12 × 10s, 7 × 60s, 16 × 300s. The average injected dose was 173.37 MBq.

#### MRI

MRI (T1-weighted) images for all the participants were acquired using a 3-Tesla Siemens Tim Trio scanner (Siemens, Erlangen, Germany) equipped with a 32-channel head coil. The T1-weighted magnetization prepared rapid gradient echo (MPRAGE) images were acquired using the following parameters: matrix size = 240 × 256, in-plane spatial resolution = 1 mm, acquisition time = 9 min 14 s, repetition time = 2300ms, echo time = 2.98ms, flip angle = 9°, inversion time = 900–1100 ms, voxel size = 1 × 1 × 1 mm^3^, 176 sagittal slices with thickness = 1.0–1.2 mm. PET and MRI data were acquired on average 2.2 ± 1.2 years apart from each other.

### Motion correction and registration

Individual total-body dynamic PET images were brain-cropped using PMOD (PMOD Technologies, LLC), and motion corrected across time frames (from frame 50, i.e., longer than 10 min of scan) using FSL’s MCFLIRT. Motion-corrected images were manually inspected to ensure consistent alignment across time frames. The 4D motion-corrected dynamic PET images were linearly (affine, with the default 12 degrees of freedom) registered to their respective T1-weighted (T1W) brain images using FSL’s FLIRT algorithm [19].

### Grey matter ROIs

Grey mater ROIs, which are known to be impacted in AD based on Braak staging [6, 17], were obtained from the cortical parcellation and subcortical segmentation (*recon-all*) of anatomical T1-weighted image using FreeSurfer (v6.0.0) [20].We focused on three subject-specific bilateral ROIs from the resulting *aseg* and *aparc* atlases [21, 22]. The ROIs were medial temporal lobe (combination of entorhinal, hippocampus, and amygdala), posterior cingulate (combination of isthmus cingulate and posterior cingulate), and lateral parietal cortex (combination of inferior parietal, superior parietal, and supramarginal) [6]. FreeSurfer ROIs from all participants were carefully inspected for quality and accuracy, particularly focusing on the grey and white matter boundaries.

Bilateral grey matter cerebellum ROIs were obtained using FreeSurfer and manually edited to remove the superior portion (using the primary fissure as the posterior boundary), given known off-target binding issues with the superior cerebellum [6]. The inferior cerebellum was used as the reference region for SUVR and distribution volume ratio (DVR) estimation. SUVR for 60–90 min was calculated as the ratio of activity in the target ROI to the mean activity in the reference region ROI (inferior cerebellum). The inferior cerebellum, instead of whole-brain grey matter, was used as a reference to avoid potential bias from off-target ^18^F-PI2620 binding in the superior cerebellar region [6]. Previous ^18^F-PI-2620 studies in aging and AD have shown no significant tau deposition in the inferior cerebellum [16, 23].

FreeSurfer was used to estimate the total (bilateral) hippocampal volume for each participant. Hippocampal volume was normalized by total intracranial volume, and its logarithm was estimated.

### Image derived input function

**Fig. 1:**
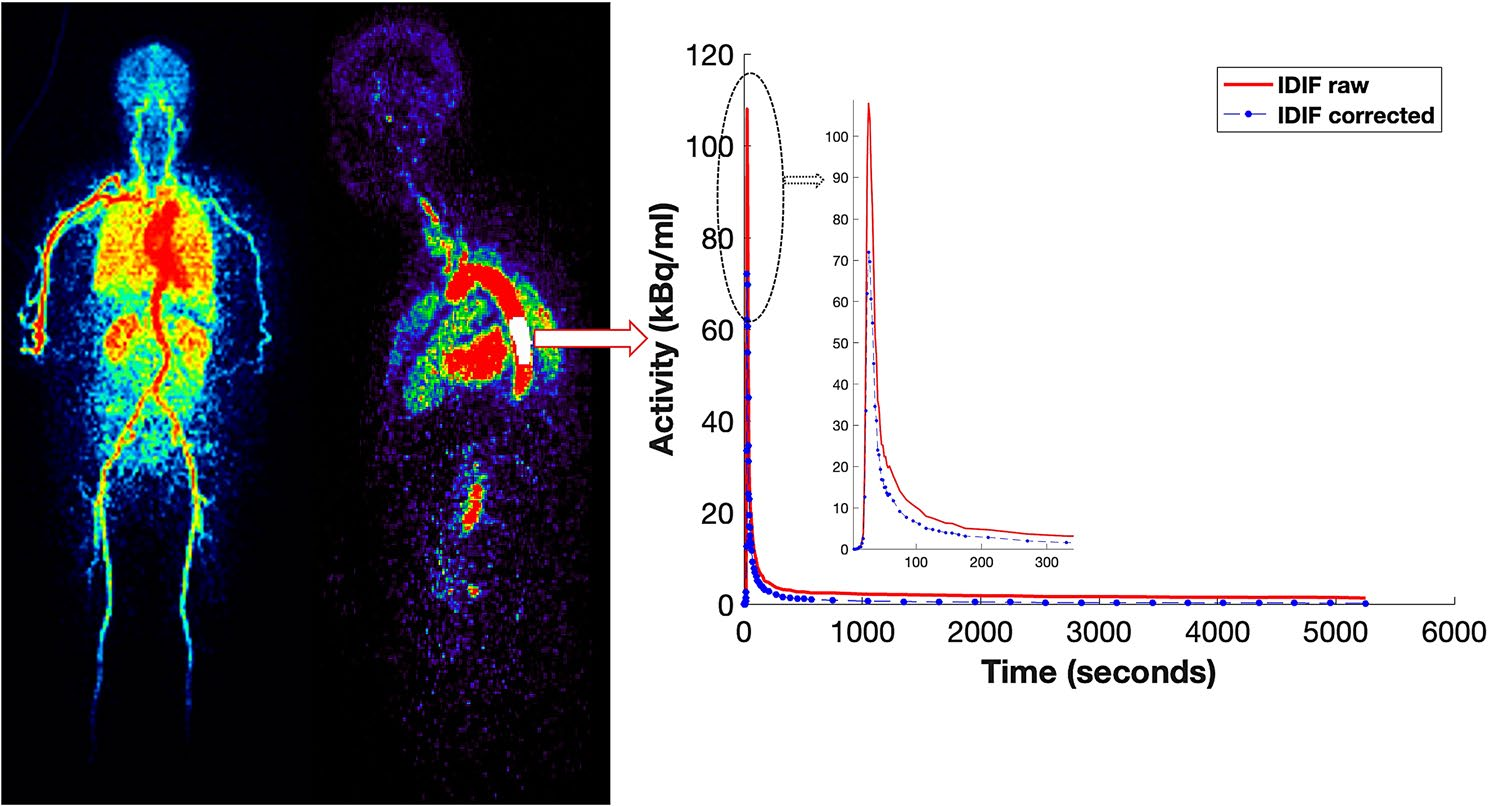
Input function derived from the descending aorta. IDIF: Image-derived input function; IDIF corrected: Plasma and metabolite corrected IDIF using population-based fractions over time. IDIF peak is zoomed for visualization

Individual image derived input functions (IDIFs) were extracted from the descending aorta using total-body dynamic PET images (Fig. 1). The descending aorta ROIs were manually drawn (mean initial frame 22.5s ± 3.8s, mean volume = 15.03 ± 5.68cm^3^) using FSLeyes image viewer, following a similar approach as employed in our previous studies [15, 24]. These manual ROIs were eroded by 1 mm in all dimensions to avoid the inclusion of vessel walls. The descending aorta time-activity curves were extracted (using PMOD) and corrected for plasma and metabolite fractions prior to kinetic modeling. Plasma correction was performed using the total plasma to whole blood ratio data acquired from a previous ^18^F-PI-2620 study investigating tau deposits in the human brain [3]. A bi-exponential function describing the parent fraction (Eq. 1), i.e. unmetabolized ^18^F-PI-2620 over time, was applied for metabolite correction [4].

1 $$p = \alpha {e^{ - {{t - {t_0}} \over {{\tau _1}}}}} + (100 - \alpha ){e^{ - {{t - {t_0}} \over {{\tau _2}}}}}$$

where *p* is percentage parent fraction; *t* is time after injection, in minutes; and α, τ_1_, τ_2_, and *t*_0_ are the model parameters (Supplement Fig. 2).

### Compartmental modeling

**Fig. 2:**
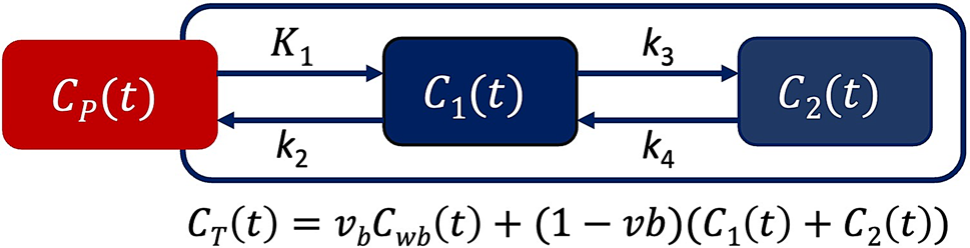
A reversible two-tissue compartmental model for ^18^F-PI-2620 kinetic modeling in the brain. C_p_(t) represents the activity of ^18^F-PI-2620 in the blood plasma. K_1_, k_2_, k_3_, and k_4_ are rate constants representing the transfer of the tracer between plasma and tissue compartments, as well as between tissue compartments. C_1_(t) and C_2_(t) represent the concentration of the tracer in brain tissue compartments. C_T_(t) represents the total ^18^F-PI-2620 activity measured by PET in the brain compartments. v_b_ is fractional blood volume, and C_wb_(t) is the tracer activity in whole blood

Dynamic time-activity curves (TACs) for each brain ROI were extracted with PMOD and fitted to a reversible two-tissue compartmental model (2TCM) (Fig. 2), using a subject-specific IDIF derived from the descending aorta. A joint estimation-based approach was used to correct for delay of tracer arrival from the input function to the tissues of interest [25]. The optimization parameters used in our modeling, such as initialization values and upper and lower limits, are summarized in Table 1.

**Table 1:** Description of optimization parameters used in kinetic modeling

Parameter	V_b_ (mL/mL)	K₁ (mL.cm⁻³.min⁻¹)	k₂ (min⁻¹)	k₃ (min⁻¹)	k₄ (min⁻¹)	Delay (s)
Optimization initials	0.01	0.01	0.01	0.01	0.01	-
Upper bound	1	1	1	1	1	20
Lower bound	0	0	0	0	0	0

The following kinetic parameters were estimated for each ROI: blood fraction volume (v_b_ [(ml/ml)]), rate constants (K_1_ [mL/cm^3^/min]), k_2_ [min^− 1^], k_3_ [min^− 1^], k_4_ [min^− 1^]), macro kinetic parameters (i.e., V_T_ [ml/cm^3^], and distribution volume ratio DVR [unitless]), and tracer arrival delay (sec) [25, 26]. The total distribution volume V_T_ was estimated as: V_T_$\mathrm{V}_{\mathrm{T}}=\frac{\mathrm{K}_1}{\mathrm{k}_2}\left(1+\frac{\mathrm{k}_3}{\mathrm{k}_4}\right).$. The DVR using the inferior cerebellum as a reference region was computed as V_T_/ V_T(ref)_, in which V_T_ represents the total distribution volume in the target ROI and V_T(ref)_ represents the total distribution volume in the reference region [3]. Additionally, SUVR corresponding to 60–90 min post injection was calculated using the inferior cerebellum as a reference region. For exploratory analysis, an additional time window of 45–75 min post injection was also considered for SUVR estimation. Additionally, to explore the accuracy and reproducibility of the kinetic parameters, we performed a sensitivity analysis by adjusting the IDIF peaks by ± 5% and ± 10% (Supplement Fig. 3).

### Logan plot analysis

Logan graphical analysis (t*=20 min) was used to estimate total distribution volume (V_T_), using the image-derived input function *C*_P_(t) and the brain time-activity curve, *C*_T_(t), for each ROI (Eq. 2) [27].

2 $${{\int_0^{\rm{t}} {{C_{\rm{T}}}} ({\rm{t}}){\rm{dt}}} \over {{C_{\rm{T}}}({\rm{t}})}} = {{\rm{V}}_{\rm{T}}}{{\int_0^{\rm{t}} {{C_{\rm{P}}}} ({\rm{t}}){\rm{dt}}} \over {{C_{\rm{T}}}({\rm{t}})}} + {\rm{ Intercept}}$$

### Scan duration

Kinetic parameters were estimated for different scan durations (from the start of injection) ranging from 10 to 90 min, in intervals of 10 min. Quantification at all ROIs were performed using dynamic data acquired at different durations and was compared with the results from the full 90-minute scan.

### Statistical analysis

Kinetic parameters (mean ± standard deviation) for all ROIs, obtained with the full 90-minute data and shorter scan durations, were calculated. Kinetic measures estimated with shorter scan durations were correlated (using Pearson’s correlation across all participants) with the respective measures estimated from the full 90-minute dynamic scan.

A linear mixed-effects model (using MATLAB’s *fitlme* function) was employed to investigate the relationship between kinetic measures (quantified with 90-minute data) and ROIs, accounting for random subject variance and the fixed effect of age. False Discovery Rate (FDR) was applied for multiple comparisons correction using the Benjamini-Hochberg procedure (using MATLAB’s *mafdr* function). Significance was determined based on the FDR-adjusted *q-value* threshold of *q* < 0.05. Additionally, as an exploratory analysis, participants’ cognitive status was incorporated as a covariate in the mixed-effects model.

Associations between V_T_ estimated using 2TCM and Logan plot analysis for each ROI were estimated using Pearson’s correlation. The significance (*p-value* at the significance level *α* = 0.05) of the association using simple linear regression (using MATLAB’s *polyfit* function) was reported. Associations between DVR and SUVR measures were estimated using Pearson’s correlation. Bilateral hippocampal volumes (logarithm of normalized volume) from MRI segmentations were also correlated with K_1_ measures of the medial temporal cortex. Additionally, a linear mixed-effects model was used to investigate the relationship between SUVR and ROIs, accounting for random subject variance and the fixed effect of age.

## Results

### Compartmental modeling

**Fig. 3:**
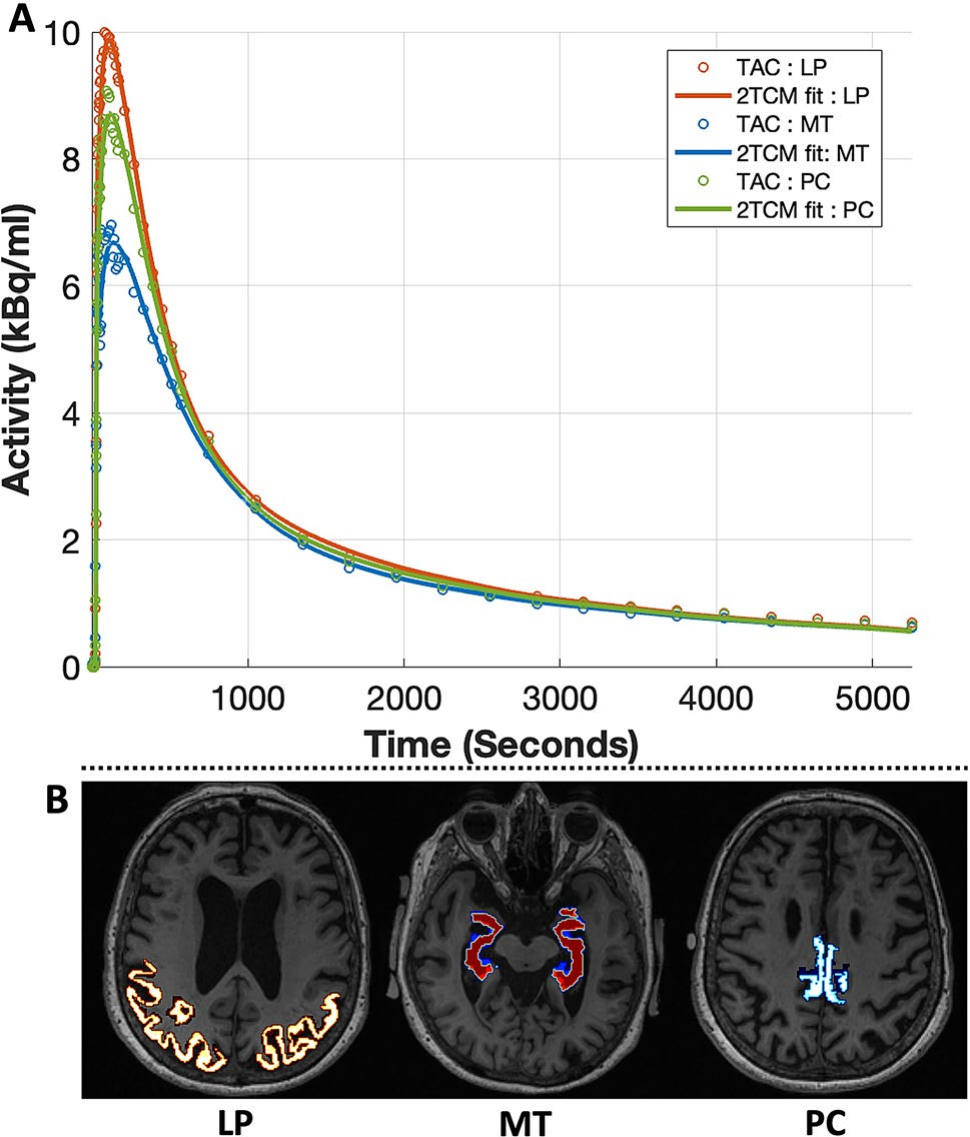
Two-tissue compartmental modeling (2TCM) curve fitting. **A**: Curve fitting for grey matter ROIs, known to be vulnerable in AD and dementia, in an individual (81-year-old female, amyloid-positive, tau-negative, diagnosed with AD). **B**: Grey matter ROIs overlaid on T1W image. TAC: Time-activity curve; LP: lateral parietal; MT: Medial Temporal; PC: Posterior cingulate

Two-tissue compartmental modeling with IDIF, corrected for plasma and metabolites, and accounting for time delay through joint estimation, demonstrated high-quality fits of ^18^F-PI-2620 binding across grey matter ROIs, for all individuals. Figure 3 displays time-activity curves for all three ROIs of an 81-year-old participant with AD, revealing strong model fits to our data.

### Regional differences in kinetic parameters

Differences in quantified kinetic parameters were observed across ROIs (Table 2). The linear mixed-effect model analyses revealed significant differences in v_b_ between LP and MT (*β* = 0.010, *q* < 0.001), and between LP and PC *(β* = 0.008, *q* < 0.001). No significant differences in V_T_ were observed across ROIs.

**Fig. 4:**
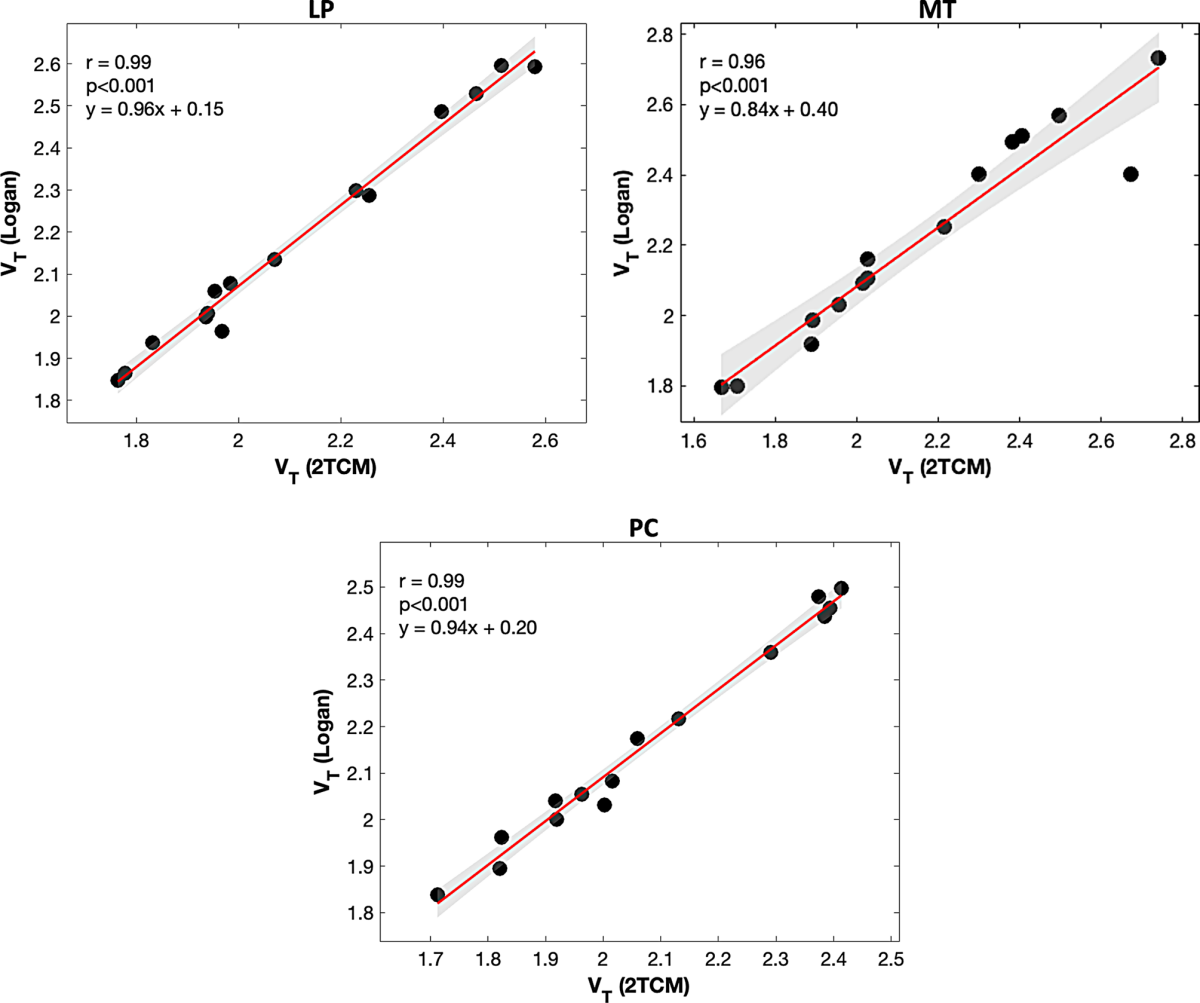
Comparison of V_T_ estimation between 2TCM and Logan analyses across ROIs. LP: Lateral parietal; MT: Medial temporal; PC: Posterior cingulate

Notably, differences in the tracer delivery parameter K_1_ were observed across ROIs. K_1_ was significantly reduced in MT relative to both LP and PC (LP and MT: *β*= -0.100, *q* < 0.001; MT and PC: *β* = 0.098, *q* < 0.001). No significant differences in K_1_ were observed between LP and PC. Similarly, significantly reduced k_2_ was observed in MT compared to LP and PC (Table 2). Furthermore, significantly reduced tracer arrival time delay was observed in MT compared to LP (*β*= -0.859, *q* < 0.001). The observed regional differences did not change when participants’ cognitive status was incorporated as a covariate in the mixed-effects model. No group differences were observed between cognitively impaired and unimpaired groups. Kinetic parameters for cognitively impaired and unimpaired groups are reported separately in the supplement (Supplement Tables 2 and 3). Sensitivity analysis in a representative cognitively impaired subject showed that, for a 5% increase or decrease in IDIF peaks, the percentage change in K_1_ and V_T_ remained within ± 0.5%. Similarly, for a 10% increase or decrease, the percentage change remained within ± 1.01%. Across all kinetic parameters, variations stayed within ± 2.5% for up to 10% manipulation of the IDIF (Supplement Table 4). This finding was consistent in a cognitively unimpaired subject as well. Excellent correlation was observed between V_T_ estimated using 2TCM and Logan plot analysis, across all ROIs (LP: *r* = 0.99, *p* < 0.001; MT: *r* = 0.96, *p* < 0.001; PC: *r* = 0.99, *p* < 0.001) (Fig. 4).

**Table 2:** Quantitative kinetic parameters across ROIs

ROIs	Kinetic parameters (mean ± std)								
	v_b_ (ml/ml)	K_1_(mL.cm⁻³.min⁻¹)	k_2_ (min⁻¹)	k_3_ (min⁻¹)	k_4_ (min⁻¹)	Delay	V_T_ (mL.cm⁻³)	DVR	V_T_Logan (mL.cm⁻³)
LP	0.029 ± 0.004*	0.372 ± 0.043	0.245 ± 0.034	0.031 ± 0.019	0.083 ± 0.055	4.625 ± 0.514	2.111 ± 0.275	1.035 ± 0.075	2.179 ± 0.266
MT	0.038 ± 0.007*	0.271 ± 0.036*	0.190 ± 0.043*	0.039 ± 0.047	0.070 ± 0.058	3.766 ± 0.902*	2.159 ± 0.331	1.057 ± 0.106	2.216 ± 0.291
PC	0.037 ± 0.007	0.369 ± 0.042	0.253 ± 0.031	0.031 ± 0.017	0.076 ± 0.038	4.323 ± 0.625	2.081 ± 0.236	1.022 ± 0.075	2.168 ± 0.225

Quantified Kinetic parameters across all 15 participants in the three key ROIs. V_T_Logan is V_T_ estimated with graphical logan plot analysis

*Significant differences at FDR *q* < 0.05 according to linear mixed-effects model analysis, comparing the measures across ROIs: *V*_b_: LP vs. MT, LP vs. PC; *K*_1_: LP vs. MT, MT vs. PC; *k*_2_: LP vs. MT, MT vs. PC; *Delay*: LP vs. MT, MT vs. PC. See supplementary material (Supplement Table 1) for full details of the results

### SUVR and its association with DVR

Significantly increased SUVR was observed in MT compared to LP and PC (LP and MT: *β* = 0.120, *p* < 0.001); MT and PC: *β*= -0.110, *p* < 0.001). Significant associations were observed between DVR and SUVR measures across all ROIs. (LP: *r* = 0.60, *p* = 0.02; MT: *r* = 0.82, *p* < 0.001; PC: *r* = 0.63, *p* = 0.01). **S**UVR using an alternative time window of 45–75 min showed a slight improvement in correlation across all ROIs (*r* = 0.69, *p* = 0.005 for LP; *r* = 0.85, *p* < 0.001 for MT; *r* = 0.72, *p* = 0.002 for PC). (Supplement Fig. 1).

### Effect of scan duration on kinetic parameters

**Fig. 5:**
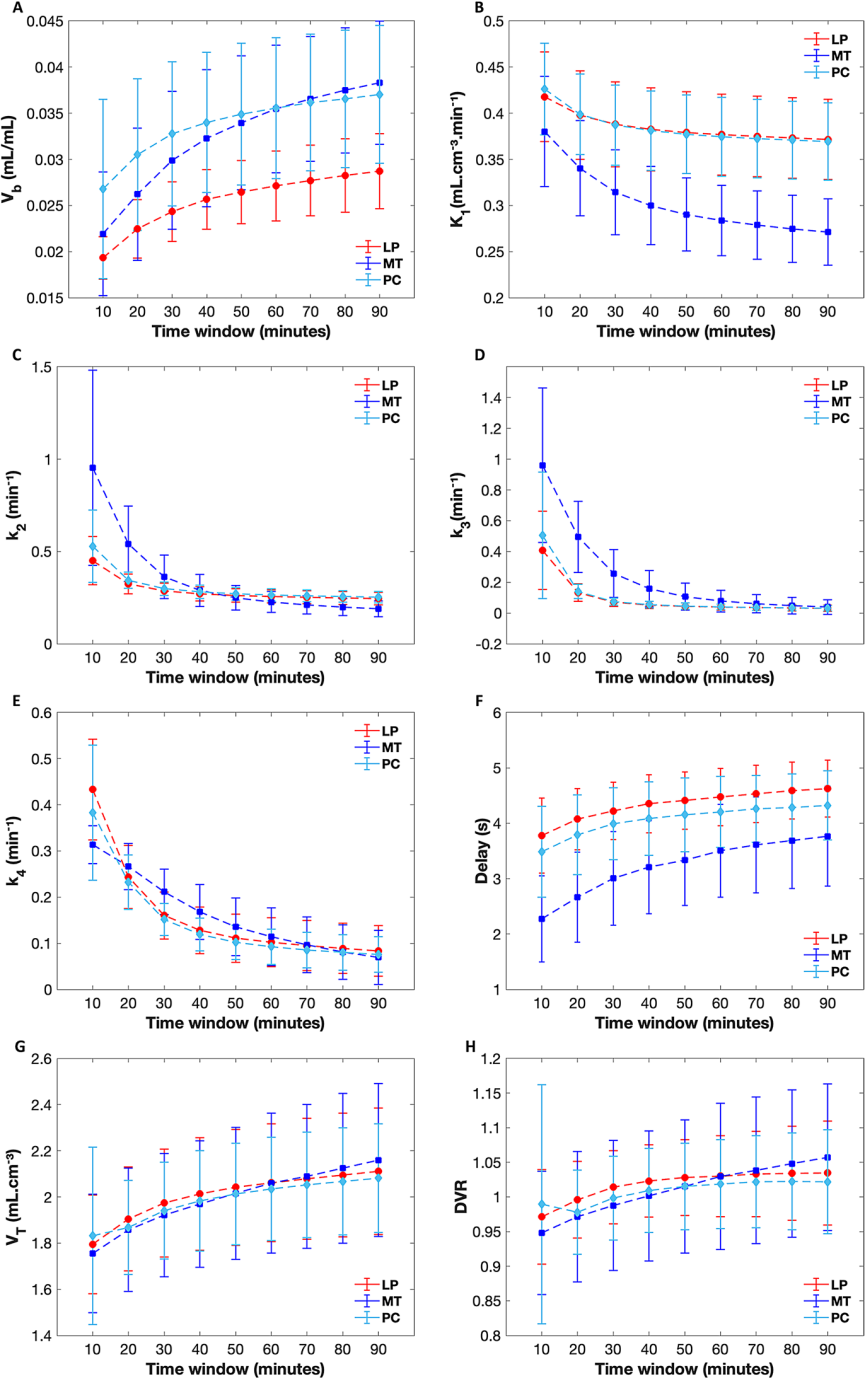
Kinetic parameters (K_1_, k_2_, k_3_, k_4_, delay, V_T_ and DVR) estimated for different scan durations. Error bars and means (connected with lines) are shown for each ROI, at each scan time. LP: Lateral Parietal; MT: Medial temporal; PC: Posterior cingulate

**Fig. 6:**
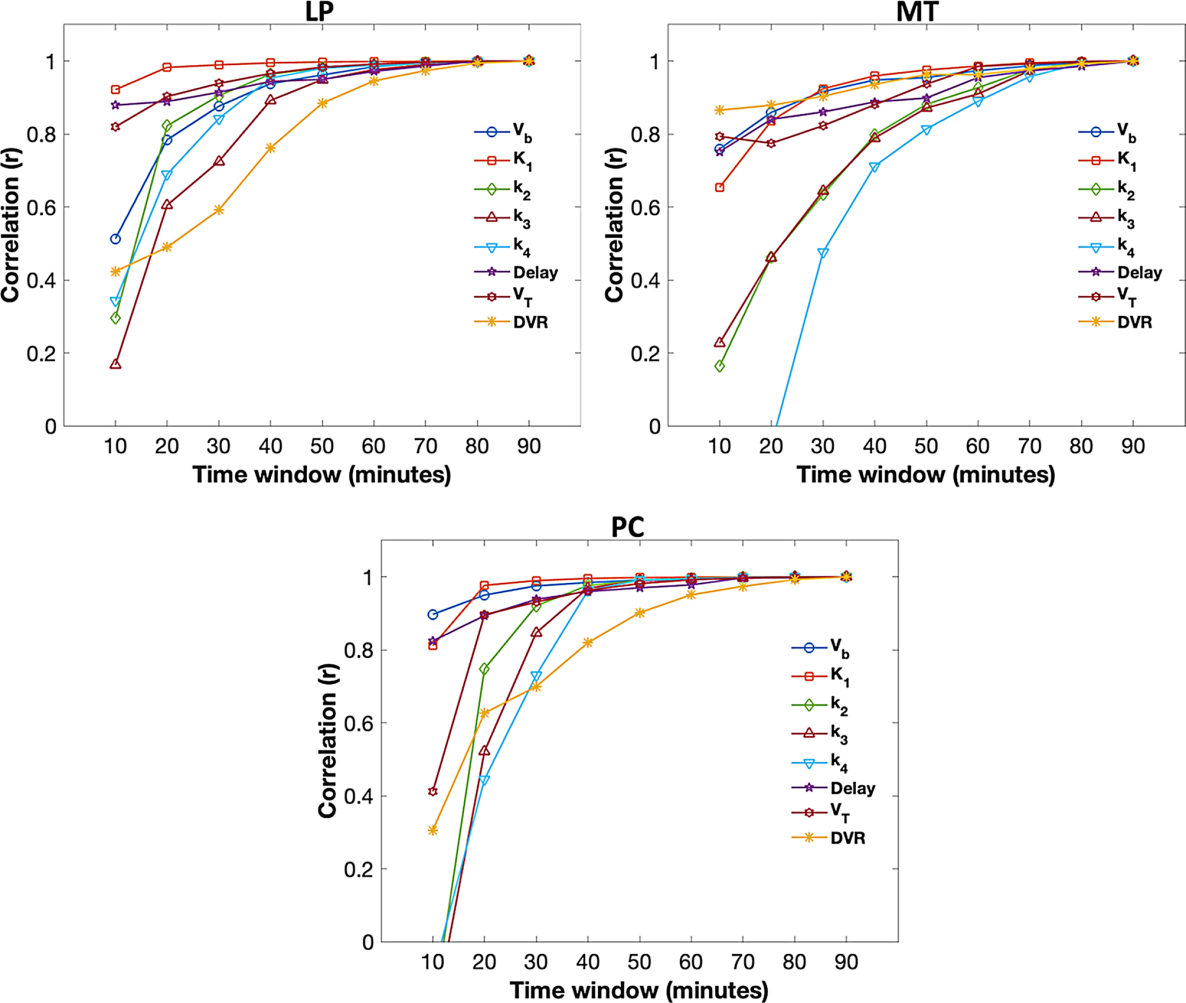
Evolution of correlations over scan time. Correlation (r) is the Pearson’s correlation between kinetic parameters estimated at each shorter time window and full 90-minute scan

All kinetic parameters remained relatively stable after the 60-minute scan window across all ROIs, demonstrating the significant correlation (*r* ≥ 0.89; *p* < 0.001) with the parameters derived from the full 90-minute data (Figs. 5 and 6). K_1_ was highly stable (*r* ≥ 0.92; *p* < 0.001) after 30 min of scan duration across all ROIs.

For PC, strong agreement (*r* ≥ 0.90; *p* < 0.001) was observed between the kinetic parameters estimated using scan durations of 50 min or longer and those derived from the full 90-minute data (Fig. 6). Similarly, for LP, strong agreements with the 90-minute data (*r* ≥ 0.88; *p* < 0.001) were observed after 50 min of scanning. For MT, compared to the other two ROIs, the parameters exhibited relatively more instability (Fig. 5). However, strong correlation (*r* ≥ 0.89; *p* < 0.001) with the full 90-minute dataset was observed after 60 min of scanning. It is noted that V_T_ and DVR (especially in MT) compared to other parameters exhibited more instability (as observed in the evolution curves) even after 60 min of scanning (Fig. 5, G, H).

### Associations between kinetic parameters

Our exploratory analyses (with linear mixed-effects model) revealed a significant association between perfusion K_1_ (*β* = 0.03, *p* = 0.012) and tracer arrival delay, including the measures computed across all ROIs. No significant associations between K_1_ and V_T_ (*β* = -0.16, *p* = 0.693) were observed. No significant correlation was observed between hippocampal volume and the K_1_ values (*r* = -0.37, *p* = 0.17) in MT.

## Discussion

This study implemented a non-invasive descending aorta IDIF, derived using a state-of-the-art total-body uEXPLORER PET/CT scanner, to quantify tau binding and tracer delivery rate from ^18^F-PI-2620 across grey matter ROIs in elderly individuals. Regional differences in tracer delivery rate were observed, suggesting medial temporal lobe to be susceptible to change in blood flow dynamics. We investigated the impact of scan duration on estimating kinetic parameters, suggesting a 60-minute scan may be required for reliable estimation of pharmacokinetic parameters (i.e., based on comparison with kinetic parameters obtained with a 90-minute scan), while a 30-minute scan may be sufficient for quantifying perfusion. A reversible 2TCM approach was used to quantify multiple micro and macro parameters, thus providing more insights into physiology. At the same time, the findings suggest that the graphical Logan plot analysis may be sufficient for estimating the macro kinetic parameter, total distribution volume, though it cannot estimate K_1_. Together, these findings highlight the utility of IDIF with total-body PET in clinical research. Given the lack of tau-positive individuals in our study, these findings need to be validated in a tau-positive cohort to assess its sensitivity in quantifying pathological tau accumulation.

This study focused on investigating tau deposition in an elderly cohort at risk of dementia. Earlier findings suggest that early tau deposition in cognitively unimpaired individuals may exhibit a divergent cortical tau PET pattern [28] and that tau PET signal in medial temporal lobe regions correlates with poorer cognitive scores in memory tests in unimpaired individuals [29]. Investigating early-stage tau PET deposition and kinetics is critical for clinical research, as well as for selecting participants for clinical trials aimed at preventing or halting disease progression. This study differed from conventional SUVR measures in tau PET because we adopted kinetic modeling for absolute and accurate quantification of micro and macro kinetic parameters, without the need for a reference region. Through kinetic modeling, our methods improves upon some limitations of SUVR, which does not account for dynamic changes in tracer distribution over time and can be impacted by choice of reference region [7]. Our kinetic modeling results of tracer binding and individual rate constants in tau-negative individuals therefore may serve as a quantitative reference for future ^18^F-PI-2620 kinetic modeling studies.

In our study, two-tissue compartmental modeling with the descending aorta IDIF demonstrated high-quality fitting of ^18^F-PI-2620 binding across grey matter ROIs. Regional differences in kinetic micro and macro parameters were observed, but no differences in total distribution volume (V_T_) were observed. Notably, reduced tracer delivery rate (K_1_) was observed in MT, which may indicate reduced perfusion in that region. Our results corroborate earlier findings that have associated hypoperfusion in the medial temporal lobes with cognitive decline, characterized by decreased performance on memory tests, and increased risk for AD [30, 31]. Hypoperfusion in the MTL, particularly in the hippocampus, has been consistently reported in arterial spin labeling (ASL) MRI studies, complementing our findings [32]. To further validate these results, future studies using a multimodal neuroimaging approach could directly compare PET-derived K_1_ measurements with cerebral blood flow (CBF) values obtained through other techniques, such as ASL MRI.

We observed reduced tracer arrival time (i.e., delay) and increased blood fraction volume in MT. We speculate that these regional differences in tracer delivery and blood flow dynamics may reflect physiological and metabolic differences in MT, which is known to be vulnerable in aging and dementia. Significant associations were also found between K_1_ and arrival delay, suggesting that potential differences in cerebral blood flow and grey matter vascular dynamics could impact both K_1_ and tracer transport. No statistically significant association was observed between K_1_ and hippocampal volume in MT. Future research with longitudinal quantitative tau PET follow-up could investigate whether structural changes in the hippocampus may impact cerebral perfusion and tau levels in healthy aging and early AD.

Not only did we observe a strong correlation between the macroparameter V_T_ as estimated by Logan graphical approach and 2TCM, the 2TCM compartment modeling in this study allowed us to estimate several microparameters, providing insight into tracer kinetics at different time windows. The Logan plot has several merits, including independence from a kinetic model, algorithmic stability, and reduced computational time [33]. Moreover, Logan plot analysis is easy to use, simple to implement, and can be useful in clinical settings for V_T_ estimation. However, it does not provide estimates of other kinetic parameters such as K_1_, k_2_, k_3_, and k_4_, which are important for understanding tracer delivery and retention mechanisms. Associations between V_T_ ​derived from 2TCM, and Logan plot analysis were strong across all ROIs. Notably, we did not observe regional variations in V_T_ measures in our cohort. Further investigation is warranted to determine if these associations are impacted in tau-positive individuals, as regional differences in tracer distribution are expected in such cases [23].

The K_1_ parameter appeared to be highly stable (compared with K_1_ estimated from the full 90 min scan) after 30 min of scan duration across all ROIs, suggesting that shorter time durations may be sufficient for the quantification of perfusion measures across grey matter ROIs. All other kinetic parameters remained relatively stable after the 60-minute scan window across all ROIs. It is important to note that kinetic parameters in MT, especially V_T_ and DVR were less stable compared to other ROIs. This is likely due to the inclusion of smaller, complex structures within the MT ROI that are more susceptible to partial volume effects and heterogeneity in tracer binding, such as the hippocampus and entorhinal cortex. An earlier study suggested that a shorter scan time (i.e., 40 min) can be sufficient for the measurements of semi-quantitative SUVR in the brain in individuals with progressive supranuclear palsy (PSP). The impact of scan duration on the estimation of quantitative kinetic microparameters has not been previously studied for this tracer. Our findings suggest that 60-minute time window may be adequate for quantifying micro and macro kinetic parameters with two-tissue compartmental modeling in elderly cohort at risk of AD. Future studies should investigate several factors that can influence the temporal evolution of kinetic parameters, including data quality, selection of ROIs, tau/amyloid status, and clinical heterogeneity.

In this study, we observed a moderate correlation between SUVR and DVR measures in the lateral parietal and posterior cingulate ROIs. Although SUVR measures with static PET do not provide absolute quantification, SUVR is a pragmatic alternative to kinetic modeling and DVR when resources and scanning time are limited. We observed regional differences in the associations between SUVR and DVR, with the association being stronger in MT compared to LT and PC. Both DVR and SUVR were relatively higher in the MT compared to other ROIs, potentially suggesting relatively higher tracer binding. We speculate that the inclusion of tau-positive individuals, i.e., those with increased tau binding in the disease epicenter, such as the MT region in AD, would result in stronger associations than those observed in our study. Interestingly, our exploratory analysis suggests that SUVR estimated using the 45–75 min window may offer a better approximation of DVR compared to the 60–90 min window, although the overall correlation remains moderate. This finding is consistent with a previous study, which reported that ^18^F-PI-2620 PET scan acquisition in AD patients between 45 and 75 min provides excellent quantification accuracy, a large effect size, and low test-retest variability [3]. However, the selection of the appropriate SUVR window across different clinical cohorts warrants further investigation.

Kinetic modeling requires an input function, which in this study was enabled by the total-body uEXPLORER PET/CT scanner to extract IDIF from larger blood pools including ventricles and aorta, which would not be possible using the conventional scanners with shorter FOV [34]. We used the descending aorta as the IDIF, which is relatively less susceptible to partial volume effects. Anatomically, the descending aorta is closer to the posterior chest wall, which protects it from cardiac and respiratory motion, making it an optimal choice for IDIF in kinetic modeling studies [35]. Furthermore, the descending aorta, instead of the ascending aorta, was selected as the IDIF in this study to avoid additional complexity and potential errors (associated with visibility of the ascending aorta in dynamic PET data) while manually extracting the ROIs. Both the left ventricle (LV) and descending aorta are viable choices for IDIF. However, earlier ^18^F-FDG kinetic modeling study comparing IDIF to the arterial input function (AIF) demonstrated that the aorta-derived IDIF remained stable before and after motion correction [36]. In contrast, the left ventricle IDIF was more affected by motion artifacts, particularly cardiac motion, leading to increased variability at later time points. Our sensitivity analysis showed that up to ± 10% manipulation of the IDIF peak values resulted in a percentage change within ± 2.5%, suggesting that the model is stable and the parameter estimates are robust to variations in the IDIF. We have previously demonstrated the utility of the descending aorta IDIF in our amyloid PET study [15].

This study also benefitted from the excellent spatial resolution (voxel size of 2.34 mm^3^) and high temporal resolution framing (2 s initial frames) of the total-body uEXPLORER PET/CT scanner. The enhanced temporal resolution enabled accurate estimation of tracer distribution in early frames. Earlier arterial blood sampling studies using ^18^F-PI-2620 had significantly lower temporal resolution, typically around 30 s [3, 4]. Higher temporal resolution could also benefit the accurate estimation of the IDIF time-activity curve. Visually, we observed a higher IDIF TAC peak (see Fig. 1) compared to the AIF TAC peak (around 20 kBq/mL in AD) reported in a previous arterial blood sampling study [3]. The temporal evolution of the activity curves from the grey matter regions were similar to those observed in our study. Expectedly, a noticeable difference was observed in our descending aorta IDIF peak compared to an earlier study utilizing the carotid artery (i.e., lower peak but varied based on the method of extraction) as an input function [23]. These variations observed in input function could impact the estimation of ^18^F-PI2620 kinetic parameters and warrant further investigation.

IDIF from total-body PET for kinetic modeling is emerging and has the potential to replace the conventional invasive arterial blood sampling approach in a long term, however, the utility of IDIF for ^18^F-PI-2620 kinetic modeling requires further investigation and validation against blood sampling. Although the arterial input function is still considered the gold standard for kinetic modeling, it requires invasive blood sampling and is also susceptible to sampling errors, delay, and dispersion [10, 23]. IDIF extraction is an alternative approach but can be very challenging when small-diameter, convoluted vessels, and complex surrounding structures are present inside the field-of-view. A recent ^18^F-PI-2620 brain study explored the utility of IDIF against AIF for estimating a macroparameter, V_T_, with the graphical Logan plot analysis [23]. However, the study utilized the carotid artery as an IDIF for the brain, which has a diameter of approximately 4 to 5 mm and is smaller than the spatial resolution of most of the clinical PET scanners (∼ 5 mm) [10]. The limited spatial resolution may introduce partial volume effects in the IDIF and may lead to incorrect parameter estimation [37]. We recommend that future studies explore the impact of IDIF selection (i.e., from other anatomical locations) in ^18^F-PI-2620 kinetic modeling studies.

^18^F-PI2620 has a high affinity for tau accumulation, which is prevalent in older individuals, even though it may not meet the criteria for tau positivity. A limitation of our tau-negative cohort was lower tau signal, especially in the early frames and smaller structures, which can contribute to lower signal-to-noise ratio (SNR). At the same time, our total-body PET scanner with higher sensitivity may address some of these SNR limitations compared to earlier PET studies. Overall, our regional average time-activity curves show similar patterns of temporal evolution when compared to earlier ^18^F- PI2620 studies [3]. While the peak and peak time may differ due to tau status and differences in temporal resolution, the evolution of the curve across all individuals was consistent and similar to previous studies. Furthermore, the SUVR range observed in this study is also comparable to earlier ^18^F-PI-2620 studies [3, 6]. These observations and the high-quality kinetic model fits in multiple brain regions (Fig. 3) reinforce the value of the observed signal and parameter estimates in this study, despite our cohort not exhibiting significant tau deposition.

There are other limitations in our study. In addition to not including tau-positive individuals, our cohort was relatively small. Our primary goal was to implement aorta-derived IDIF in kinetic modeling; however, there is an avenue for future research to apply our approach to investigate differences in grey matter tracer dynamics between tau-positive and tau-negative individuals in a larger cohort. The absence of arterial blood sampling limits our ability to validate the aorta-derived IDIF against a true plasma input function. We did not consider the potential intersubject variability in ^18^F-PI-2620 metabolism for metabolite correction of our IDIF curves. ^18^F-PI-2620 metabolism may vary with the age or cognitive status, as slightly faster metabolism has been reported in AD compared to healthy subjects [3]. This study implemented a bi-exponential function, as discussed in a previous study [4], to estimate the parent fraction for metabolite correction. A full examination of potential individual differences in ^18^F-PI-2620 metabolism, especially among aging, mild cognitive impairment, AD, and control individuals, would facilitate a more accurate quantification and interpretation of tracer dynamics in the brain.

## Conclusion

This study implemented a descending aorta IDIF, derived using a novel total-body uEXPLORER PET/CT scanner, for the non-invasive quantification of ^18^F-PI-2620 in the brain of elderly individuals at risk of AD. The study used a reversible 2TCM and a graphical Logan plot analysis to quantify tracer kinetics. No significant differences in regional total distribution volume were observed, but there was a significant reduction in the tracer delivery rate, K_1_, in MT. Our findings suggest that a 60-minute scan window may be required for the reliable quantification of kinetic parameters using IDIF, whereas a 30-minute scan time may be sufficient for the quantification of K_1_. These findings need to be validated in a larger tau-positive AD cohort. Overall, the study demonstrated the utility of the uEXPLORER PET/CT scanner and kinetic modeling with IDIF in clinical research.

## Electronic supplementary material

Below is the link to the electronic supplementary material.

Supplementary Material 1

## Data Availability

The data from this study are not publicly available due to the privacy issues of the participants. Data will be shared upon reasonable request for research only after ethical approval for the specific project.
