# Supplementary material for: Kinetic modeling of 18F-PI-2620 binding in the brain using an image-derived input function with total-body PET
Source: EJNMMI Res. 2025 May 30;15:62. doi: 10.1186/s13550-025-01260-4 (PMC12125444; doi:10.1186/s13550-025-01260-4)
Supplement: Supplementary file 1 — Supplementary Material 1 [file 13550_2025_1260_MOESM1_ESM.docx]

**Kinetic modeling of ^18^F-PI-2620 binding in the brain using an image-derived input function with total-body PET**

Anjan Bhattarai^1,2*^, Emily Nicole Holy^1,2^, Yiran Wang^2,3^, Benjamin A. Spencer^3^, Guobao Wang^3^, Charles DeCarli^1^, Audrey P. Fan^1,2^

^1^ Department of Neurology, University of California, Davis, Davis, CA, USA

^2^ Department of Biomedical Engineering, University of California, Davis, Davis, CA, USA

^3^ Department of Radiology, University of California, Davis, Davis, CA, USA

Corresponding author information:

* Correspondence to: Anjan Bhattarai; Department of Neurology and Department of Biomedical Engineering, University of California, Davis, 1590 Drew Avenue, Unit #100 Davis, CA 95618, USA.

E-mail: [anbhattarai@ucdavis.edu](mailto:anbhattarai@ucdavis.edu)

**Supplement Table 1:** Relationship between kinetic measures (quantified with 90-minute data) and ROIs, accounting for random subject variance and the fixed effect of age, estimated using a linear mixed-effects model.

| **Measure** | **Comparison** | **Estimate** | **SE** | **tStat** | **DF** | **pValue** | **qValue** | **Lower CI** | **Upper CI** |
| --- | --- | --- | --- | --- | --- | --- | --- | --- | --- |
| **v_b_** | LP and MT | 0.010 | 0.001 | 6.591 | 41 | <0.001 | <0.001* | 0.007 | 0.013 |
|  | LP and PC | 0.008 | 0.001 | 5.714 | 41 | <0.001 | <0.001* | 0.005 | 0.011 |
|  | MT and PC | -0.001 | 0.001 | -0.877 | 41 | 0.386 | 0.518 | -0.004 | 0.002 |
| **K_1_** | LP and MT | -0.100 | 0.009 | -11.316 | 41 | <0.001 | <0.001* | -0.118 | -0.082 |
|  | LP and PC | -0.002 | 0.009 | -0.259 | 41 | 0.797 | 0.832 | -0.020 | 0.016 |
|  | MT and PC | 0.098 | 0.009 | 11.057 | 41 | <0.001 | <0.001* | 0.080 | 0.116 |
| **k_2_** | LP and MT | -0.055 | 0.008 | -6.799 | 41 | <0.001 | <0.001* | -0.071 | -0.039 |
|  | LP and PC | 0.008 | 0.008 | 0.968 | 41 | 0.339 | 0.518 | -0.009 | 0.024 |
|  | MT and PC | 0.063 | 0.008 | 7.768 | 41 | <0.001 | <0.001* | 0.047 | 0.079 |
| **k_3_** | LP and MT | 0.009 | 0.010 | 0.914 | 41 | 0.366 | 0.518 | -0.011 | 0.028 |
|  | LP and PC | 0.000 | 0.010 | 0.041 | 41 | 0.967 | 0.967 | -0.019 | 0.020 |
|  | MT and PC | -0.008 | 0.010 | -0.872 | 41 | 0.388 | 0.518 | -0.028 | 0.011 |
| **k_4_** | LP and MT | -0.014 | 0.014 | -0.965 | 41 | 0.340 | 0.518 | -0.043 | 0.015 |
|  | LP and PC | -0.008 | 0.014 | -0.527 | 41 | 0.601 | 0.687 | -0.0369 | 0.022 |
|  | MT and PC | 0.006 | 0.014 | 0.438 | 41 | 0.664 | 0.724 | -0.023 | 0.036 |
| **Delay** | LP and MT | -0.859 | 0.158 | -5.440 | 41 | <0.001 | <0.001* | -1.178 | -0.540 |
|  | LP and PC | -0.303 | 0.158 | -1.916 | 41 | 0.062 | 0.166 | -0.622 | 0.016 |
|  | MT and PC | 0.557 | 0.158 | 3.524 | 41 | 0.001 | 0.003* | 0.238 | 0.876 |
| **V_T_** | LP and MT | 0.048 | 0.051 | 0.953 | 41 | 0.346 | 0.518 | -0.054 | 0.151 |
|  | LP and PC | -0.030 | 0.051 | -0.586 | 41 | 0.561 | 0.687 | -0.132 | 0.073 |
|  | MT and PC | -0.078 | 0.051 | -1.540 | 41 | 0.131 | 0.305 | -0.180 | 0.024 |
| **DVR** | LP and MT | 0.023 | 0.023 | 0.960 | 41 | 0.343 | 0.518 | -0.025 | 0.070 |
|  | LP and PC | -0.013 | 0.023 | -0.545 | 41 | 0.589 | 0.687 | -0.060 | 0.035 |
|  | MT and PC | -0.035 | 0.023 | -1.505 | 41 | 0.140 | 0.305 | -0.083 | 0.012 |

Significance was determined based on the FDR-adjusted q-value threshold of q < 0.05. Standard error (SE). t-statistics (tStat). Degree of freedom (DF). Confidence interval (CI).


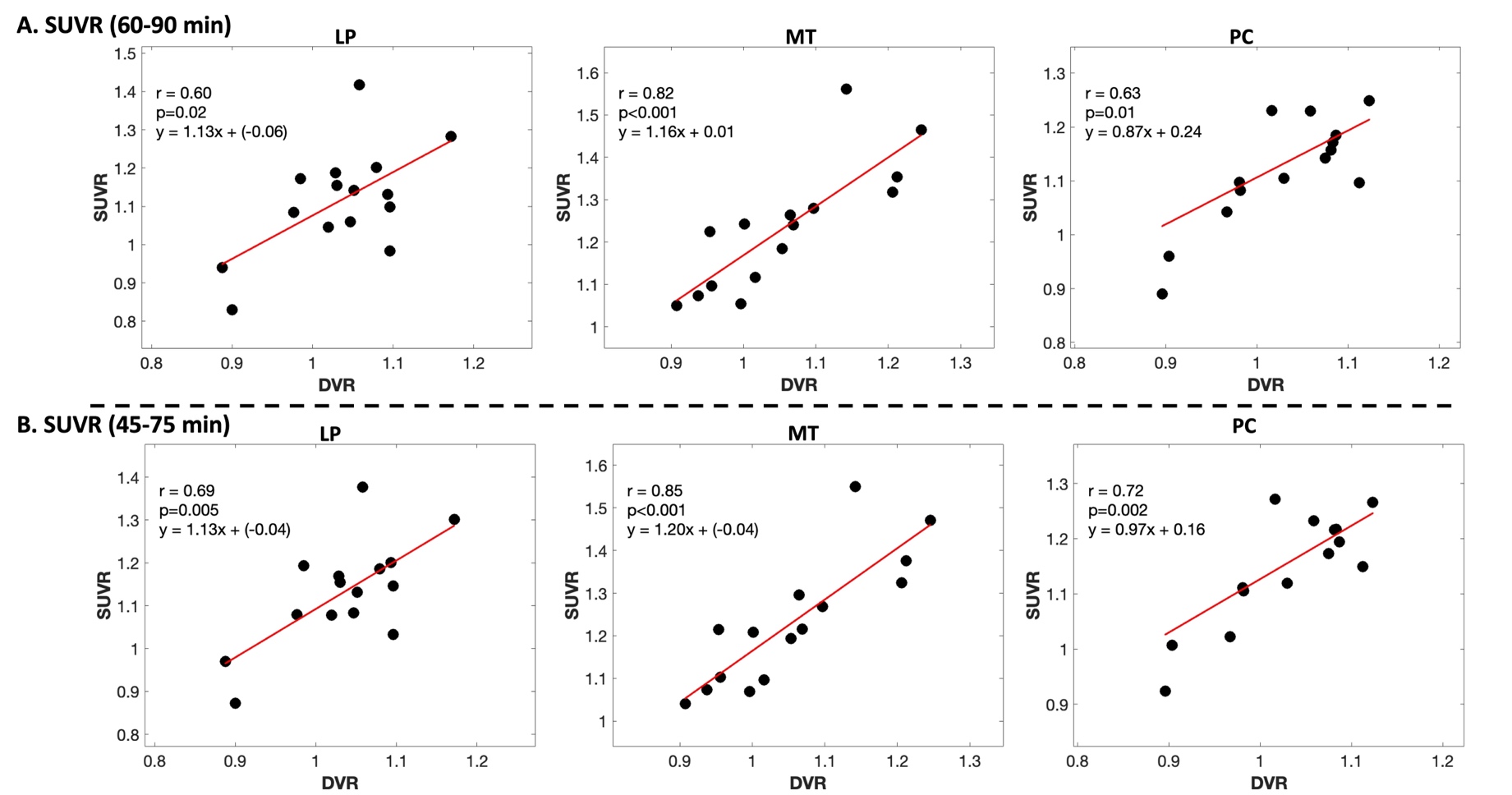


**Supplement Figure 1:** Association between SUVR (Panel A: from 60 to 90 min; Panel B: from 45 to 75 min) and DVR measures across ROIs. The inferior grey matter cerebellum was used as the reference region for SUVR and DVR estimation.


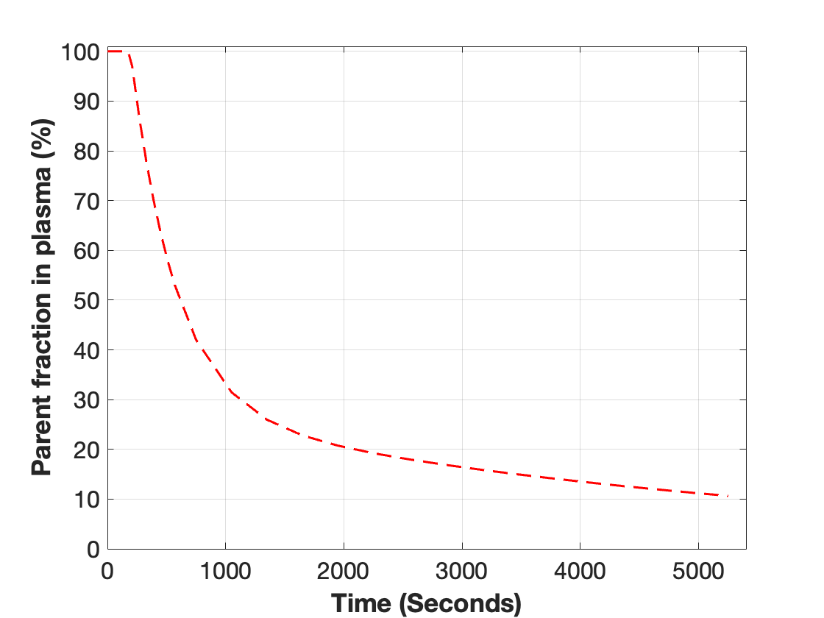


**Supplement Figure 2:** Illustrative example of the parent fraction in venous plasma over time was described with a biexponential function with the following parameters: α=72.01, τ_1_=6.38 min, τ_2_=87.15 min, t_0_ = 3.23 min, as discussed by Mueller et al.,2020 [4].

| **ROIs** | **Kinetic parameters (mean± std) in cognitively unimpaired individuals** | | | | | | | | |
| --- | --- | --- | --- | --- | --- | --- | --- | --- | --- |
|  | **v_b_ (ml/ml)** | **K_1_(mL.cm⁻³.min⁻¹)** | **k_2_ (min⁻¹)** | **k_3_ (min⁻¹)** | **k_4_ (min⁻¹)** | **Delay** | **V_T_ (mL.cm⁻³)** | **DVR** | **V_T_Logan (mL.cm⁻³)** |
| **LP** | 0.028 ± 0.004 | 0.358 ± 0.039 | 0.247 ± 0.037 | 0.036 ± 0.020 | 0.094 ± 0.061 | 4.501 ± 0.412 | 2.053 ± 0.224 | 1.025 ± 0.075 | 2.122 ± 0.220 |
| **MT** | 0.039 ± 0.006 | 0.261 ± 0.029 | 0.189 ± 0.046 | 0.036 ± 0.037 | 0.065 ± 0.049 | 3.636 ± 0.904 | 2.120 ± 0.302 | 1.055 ± 0.090 | 2.178 ± 0.253 |
| **PC** | 0.039 ± 0.008 | 0.367 ± 0.044 | 0.259 ± 0.033 | 0.034 ± 0.017 | 0.080 ± 0.040 | 4.208 ± 0.559 | 2.060 ± 0.225 | 1.029 ± 0.080 | 2.153 ± 0.215 |

**Supplement Table 2:** Quantitative Kinetic parameters in 11 cognitively unimpaired individuals.

V_T_Logan is V_T_ estimated with graphical Logan plot analysis.

| **ROIs** | **Kinetic parameters (mean± std) in cognitively impaired individuals** | | | | | | | | |
| --- | --- | --- | --- | --- | --- | --- | --- | --- | --- |
|  | **v_b_ (ml/ml)** | **K_1_(mL.cm⁻³.min⁻¹)** | **k_2_ (min⁻¹)** | **k_3_ (min⁻¹)** | **k_4_ (min⁻¹)** | **Delay** | **V_T_ (mL.cm⁻³)** | **DVR** | **V_T_Logan (mL.cm⁻³)** |
| **LP** | 0.031 ± 0.005 | 0.408 ± 0.035 | 0.238 ± 0.027 | 0.017 ± 0.005 | 0.054 ± 0.018 | 4.968 ± 0.673 | 2.272 ± 0.371 | 1.061 ± 0.080 | 2.334 ± 0.353 |
| **MT** | 0.036 ± 0.008 | 0.298 ± 0.042 | 0.192 ± 0.039 | 0.050 ± 0.076 | 0.080 ± 0.087 | 4.123 ± 0.914 | 2.267 ± 0.432 | 1.062 ± 0.159 | 2.323 ± 0.401 |
| **PC** | 0.033 ± 0.006 | 0.375 ± 0.042 | 0.235 ± 0.016 | 0.022 ± 0.010 | 0.065 ± 0.034 | 4.638 ± 0.776 | 2.140 ± 0.293 | 1.003 ± 0.064 | 2.210 ± 0.280 |

**Supplement Table 3:** Quantitative Kinetic parameters in 4 cognitively impaired individuals.


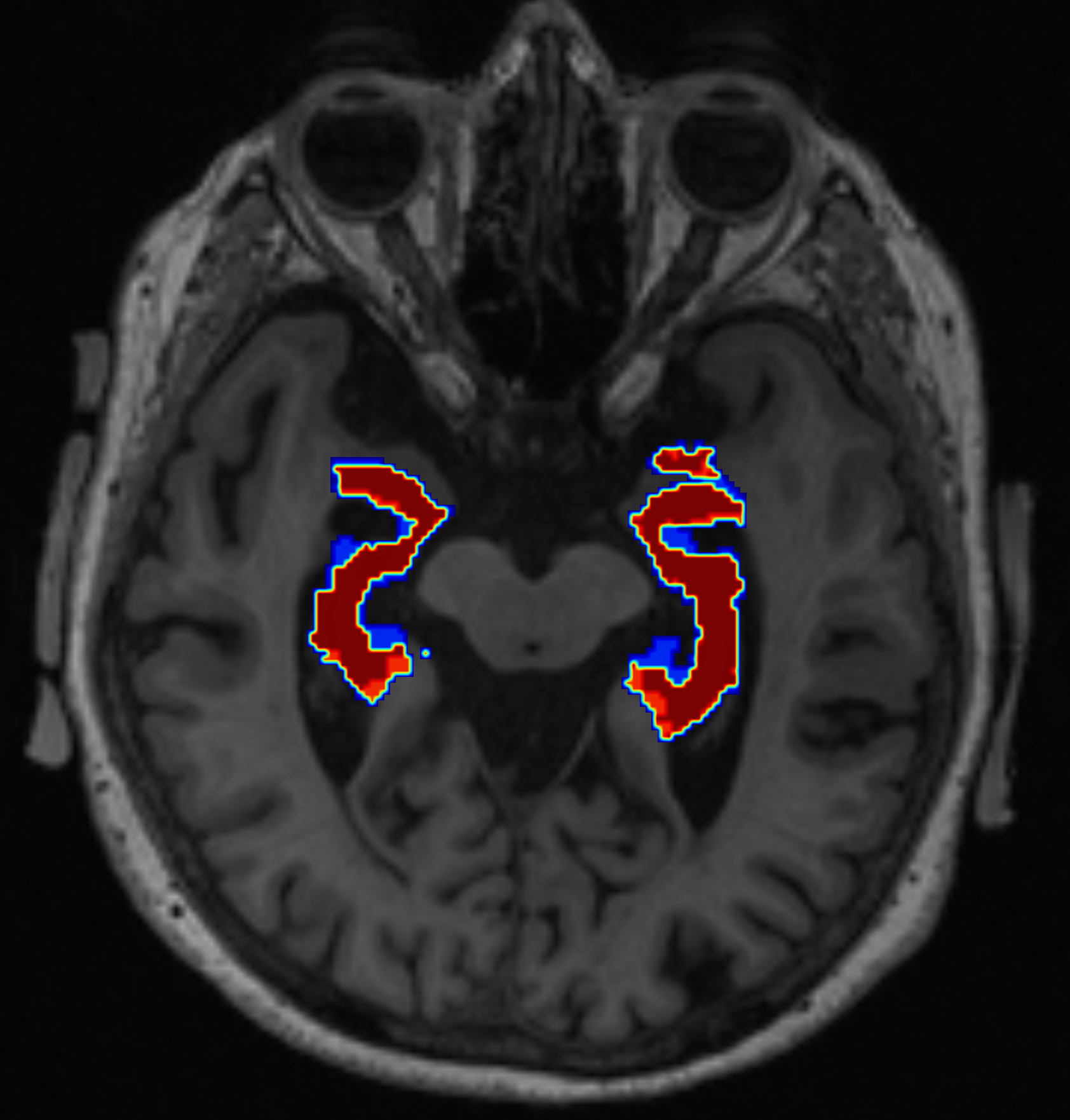

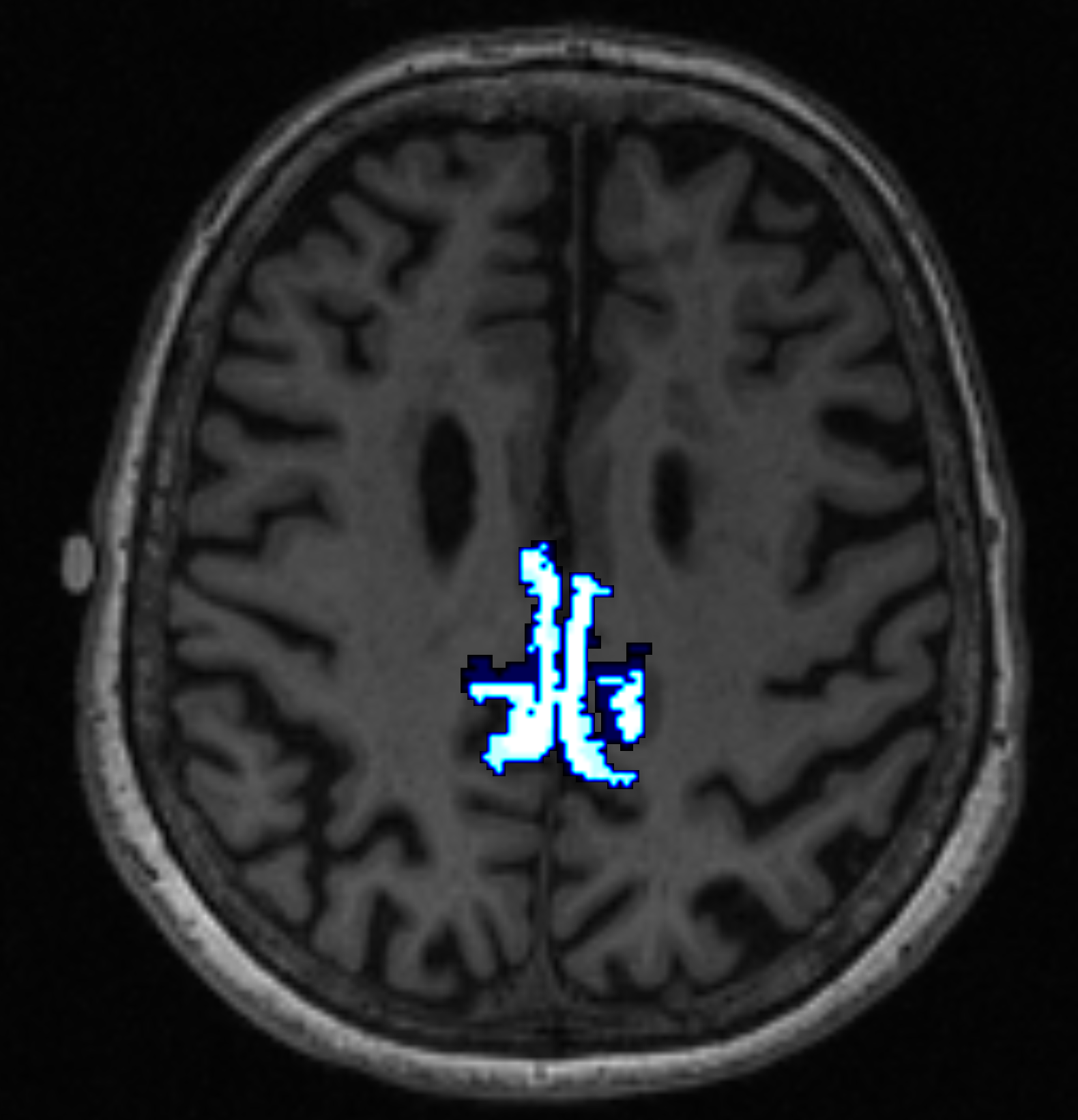

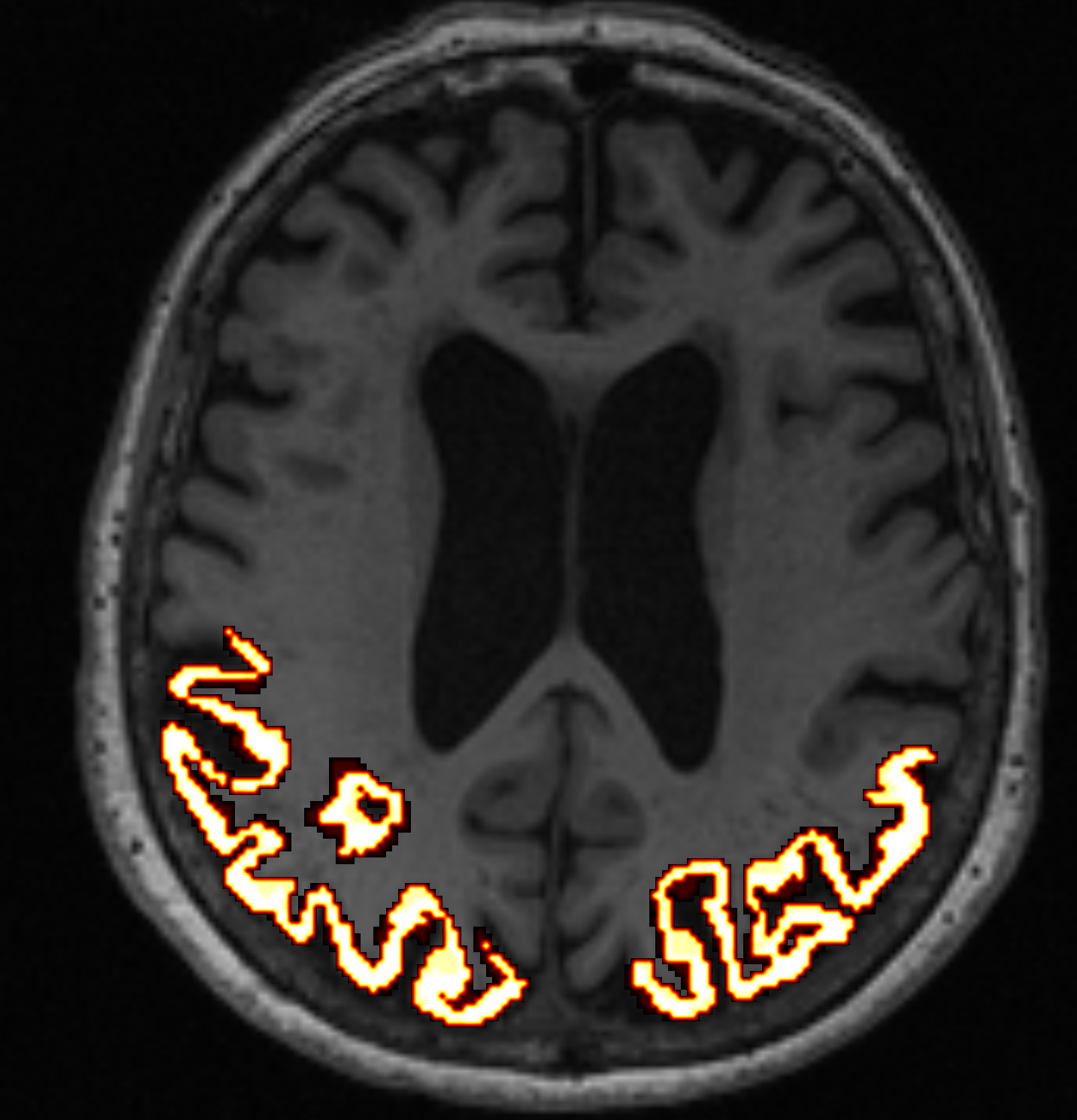


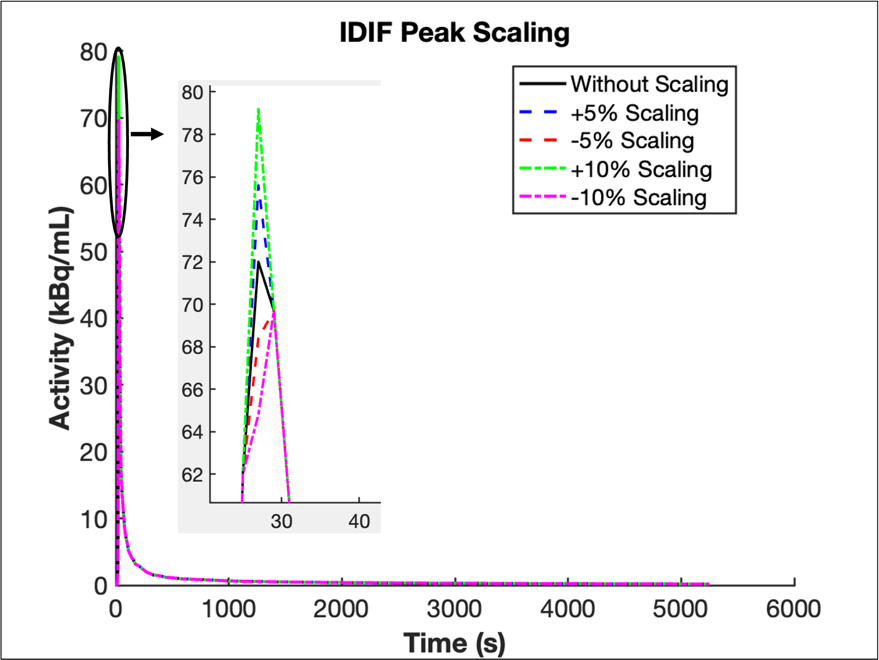


**Supplement Figure 3**: IDIF peak scaling for sensitivity analysis in a representative subject (81-year-old female, amyloid-positive, tau-negative, diagnosed with AD).

**Supplement Table 4:** IDIF peak variation and its impact on kinetic parameters in a representative subject (81-year-old female, amyloid-positive, tau-negative, diagnosed with AD).

| **Parameters** | **No Scaling** | **With +5% Scaling** | | **With -5% Scaling** | | **With +10% Scaling** | | **With -10% Scaling** | |
| --- | --- | --- | --- | --- | --- | --- | --- | --- | --- |
|  | **Values** | **Values** | **% Change** | **values** | **% Change** | **values** | **% Change** | **values** | **% Change** |
| **ROI: LP** | | | | | | | | | |
| **vb** | 0.035 | 0.034 | -1.092 | 0.035 | 1.098 | 0.034 | -2.178 | 0.036 | 2.201 |
| **K_1_** | 0.449 | 0.446 | -0.493 | 0.451 | 0.499 | 0.444 | -0.981 | 0.453 | 1.003 |
| **k_2_** | 0.248 | 0.247 | -0.347 | 0.249 | 0.355 | 0.246 | -0.689 | 0.250 | 0.713 |
| **k_3_** | 0.022 | 0.022 | -0.694 | 0.022 | 0.717 | 0.021 | -1.365 | 0.022 | 1.457 |
| **k_4_** | 0.056 | 0.055 | -0.794 | 0.056 | 0.812 | 0.055 | -1.570 | 0.057 | 1.646 |
| **Delay** | 4.900 | 4.880 | -0.408 | 4.920 | 0.408 | 4.860 | -0.816 | 4.940 | 0.816 |
| **V_T_** | 2.514 | 2.511 | -0.119 | 2.516 | 0.115 | 2.508 | -0.235 | 2.519 | 0.235 |
| **DVR** | 1.030 | 1.030 | 0.024 | 1.030 | -0.028 | 1.030 | 0.0320 | 1.029 | -0.056 |
| **ROI: MT** | | | | | | | | | |
| **vb** | 0.030 | 0.030 | -0.745 | 0.030 | 0.752 | 0.030 | -1.486 | 0.031 | 1.506 |
| **K_1_** | 0.271 | 0.270 | -0.435 | 0.273 | 0.439 | 0.269 | -0.870 | 0.274 | 0.884 |
| **k_2_** | 0.161 | 0.161 | -0.285 | 0.162 | 0.291 | 0.161 | -0.570 | 0.162 | 0.582 |
| **k_3_** | 0.012 | 0.012 | -0.526 | 0.012 | 0.543 | 0.011 | -1.035 | 0.012 | 1.096 |
| **k_4_** | 0.036 | 0.036 | -0.729 | 0.037 | 0.746 | 0.036 | -1.445 | 0.037 | 1.511 |
| **Delay** | 2.98 | 2.97 | -0.336 | 2.990 | 0.336 | 2.960 | -0.671 | 3.000 | 0.671 |
| **V_T_** | 2.214 | 2.212 | -0.099 | 2.217 | 0.099 | 2.210 | -0.203 | 2.219 | 0.199 |
| **DVR** | 0.907 | 0.908 | 0.044 | 0.907 | -0.044 | 0.908 | 0.063 | 0.907 | -0.092 |
| **ROI (PC)** | | | | | | | | | |
| **vb** | 0.026 | 0.025 | -1.321 | 0.026 | 1.156 | 0.025 | -2.461 | 0.026 | 2.489 |
| **K_1_** | 0.381 | 0.380 | -0.472 | 0.383 | 0.477 | 0.378 | -0.941 | 0.385 | 0.960 |
| **k_2_** | 0.217 | 0.216 | -0.318 | 0.218 | 0.327 | 0.216 | -0.636 | 0.219 | 0.654 |
| **k_3_** | 0.017 | 0.017 | -0.589 | 0.018 | 0.612 | 0.017 | -1.166 | 0.018 | 1.235 |
| **K_4_** | 0.048 | 0.048 | -0.736 | 0.049 | 0.752 | 0.048 | -1.455 | 0.049 | 1.523 |
| **Delay** | 3.850 | 3.830 | -0.519 | 3.860 | 0.260 | 3.820 | -0.779 | 3.88 | 0.779 |
| **V_T_** | 2.393 | 2.391 | -0.113 | 2.396 | 0.117 | 2.388 | -0.226 | 2.399 | 0.230 |
| **DVR** | 0.981 | 0.981 | 0.031 | 0.981 | -0.026 | 0.981 | 0.041 | 0.98 | -0.061 |

The IDIF peaks were adjusted by ±5% and ±10%, and the impact on kinetic parameters was assessed.
